# Microenvironments of tuberculous granuloma: advances and opportunities for therapy

**DOI:** 10.3389/fimmu.2025.1575133

**Published:** 2025-03-24

**Authors:** Gesa Krueger, Shah Faisal, Anca Dorhoi

**Affiliations:** ^1^ Institute of Immunology, Friedrich-Loeffler-Institut, Federal Research Institute for Animal Health, Greifswald, Germany; ^2^ Institute of Medical Biochemistry and Molecular Biology, University Medicine Greifswald, Greifswald, Germany; ^3^ Faculty of Mathematics and Natural Sciences, University of Greifswald, Greifswald, Germany

**Keywords:** tuberculosis, granuloma, spatial biology, host-directed therapy, immunity

## Abstract

The hallmark tissue lesions of tuberculosis (TB) are granulomas. These multicellular structures exhibit varying degrees of cellular complexity, are dynamic, and show considerable diversity within and between hosts. Categorization based on gross pathologic features, particularly caseation and necrosis, was historically coined prior to the identification of mycobacteria as the causative agent of TB. More recently, granuloma zonation based on immune cell composition, metabolite abundance, and physical characteristics has gained attention. With the advent of single-cell analyses, distinct microenvironments and cellular ecosystems within TB granulomas have been identified. We summarize the architecture of TB granulomas and highlight their cellular heterogeneity, including cell niches as well as physical factors such as oxygen gradients that modulate lesion fate. We discuss opportunities for therapy, highlighting new models and the power of *in silico* modeling to unravel granuloma features and trajectories. Understanding the relevance of the granuloma microenvironment to disease pathophysiology will facilitate the development of more effective interventions, such as host-directed therapies for TB.

## Introduction

Tuberculosis (TB) is a major global health threat (1). TB is caused by *Mycobacterium tuberculosis* (Mtb), which usually infects the lungs. The pathological hallmark of TB and the center of bacteria-host interaction are the tuberculous granulomas. These are organized cellular structures generated by the host’s immune response in order to contain Mtb (2). However, the bacteria may override the immune control in these lesions (3). Granulomas are highly heterogenous, comprising microenvironments with diverse cellular compositions and various biochemical characteristics including gas, metabolite and nutrient gradients (4, 5). Such heterogeneity profoundly influences host immunity and Mtb biology, affecting disease progression, latency, and potential reactivation.

Mtb actively drives granuloma biogenesis to its own benefit (6). Accordingly, granuloma characteristics are relevant to both TB chemotherapy and host-directed therapies (HDTs) (7, 8). Current treatment regimens for drug-sensitive TB involve a prolonged course of multiple antibiotics, which poses challenges in terms of adherence and efficacy. The emergence of drug-resistant TB further complicates the treatment. Given the limitations of conventional chemotherapy and the threat of increasing drug resistance, HDTs have emerged as a promising alternative (1). HDTs aim to enhance the host immune response to effectively eliminate Mtb or limit deleterious inflammation, potentially shortening treatment duration, preventing the development of resistance, and improving the efficacy of existing anti-TB drugs. This review focuses on microenvironments within pulmonary TB granulomas, specifically zonation, molecular gradients, and cellular ecosystems revealed by spatial biology. We discuss the role of microenvironments in disease pathogenesis and highlight their relevance for HDT against TB.

## The manifolds of TB granuloma heterogeneity

### Cellular zonation of granulomas

**Figure 1:**
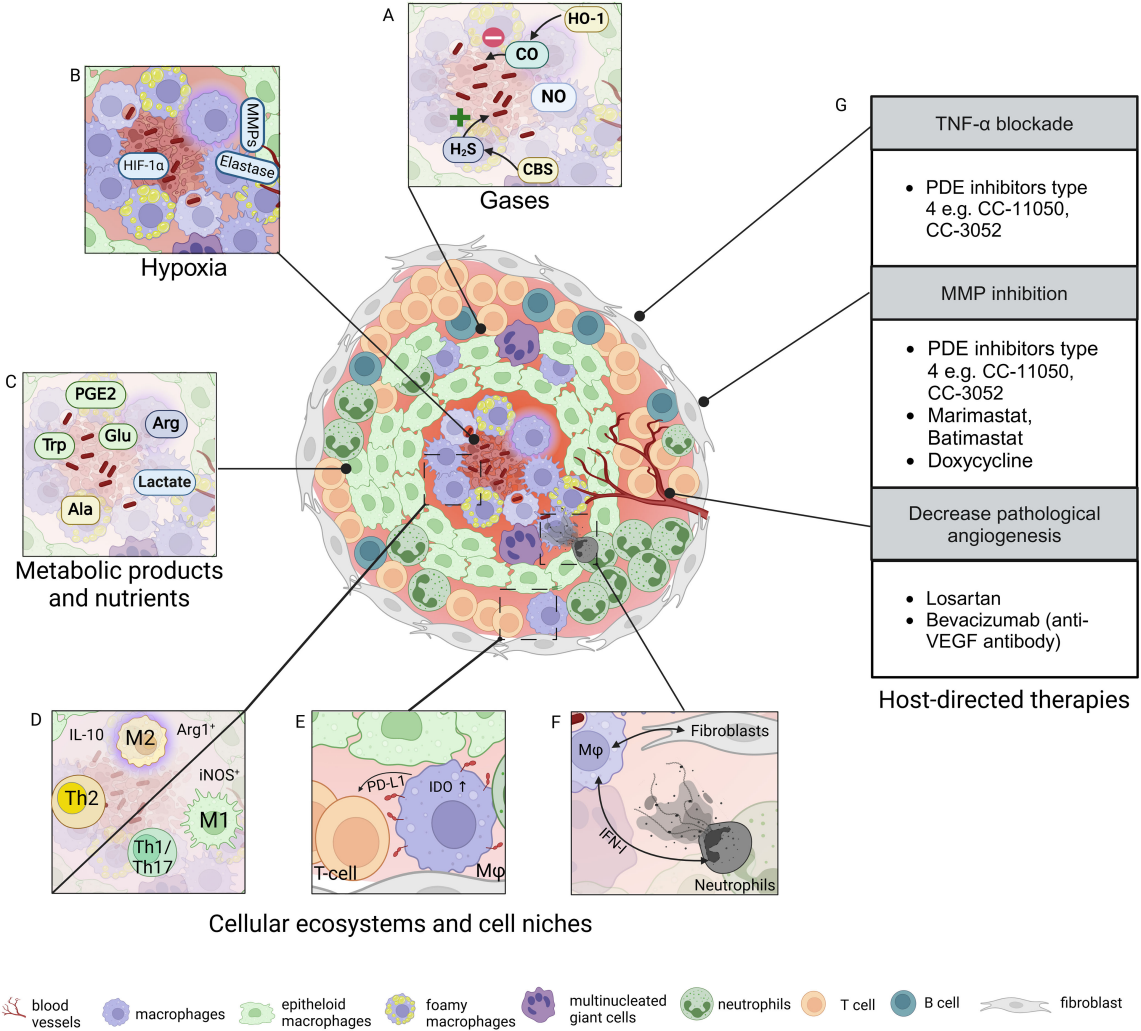
Tuberculous granuloma microenvironment and host-directed therapies. Granulomas are hallmark lesions of tuberculosis (TB) and exhibit distinct cellular zonation, gradients of gases such as oxygen or carbon monoxide **(A, B)**, nutrients such as amino acids and glucose, and various metabolites **(C)**. **(D-F)** Distinct cellular ecosystems have been identified in TB granulomas. These are defined by polarized macrophages and T-cell immune responses, distinct features of immune cells including transformed macrophages, and cell clusters with distinct spatial positioning. **(G)** Approaches for host-directed therapies (HDTs) targeting granuloma biology include modulation of inflammation, tissue matrix, and neovascularization. Ala, alanine; Arg, arginine; CBS, cystathionine β-synthase; CO, carbon monoxide; H_2_S, hydrogen sulfide; Glu, glucose; HIF, hypoxia-inducible factor; HO-1, hemeoxygenase-1; IDO, indoleamine dioxygenase; IFN, interferon; IL, interleukin; iNOS, inducible nitric oxide synthase; Mφ, macrophage; MMP, matrix metalloprotease; NO, nitric oxide; PDE, phosphodiesterase; PD-L1, programmed cell death ligand 1; PGE, prostaglandin; T, T-cell; Th, T-helper cell; TNF, tumor necrosis factor; Trp, tryptophan; VEGF, vascular endothelial growth factor. Figure created with Biorender.

The granuloma as a histopathologic hallmark of TB was described already in the 19^th^ century (9, 10). Prior to the introduction of TB chemotherapy, knowledge of the granuloma was obtained from autopsies. Today, animal models recapitulate the complexity of TB granulomas to varying degrees (8). Mouse models contributed greatly to the understanding of immune responses in TB, however usually lack organized granulomas, cavitation and fibrosis (11). Natural TB hosts such as cattle, pigs and non-human primates (NHPs) present well-organized and histopathological diverse lung granulomas which resemble lesions in humans (12, 13). Guinea pigs (14) and rabbits (15) develop necrotic lesions and cavities, yet the immunological toolbox is restricted for these species. Thus, spatial insights about TB granulomas emerge from few mammalian TB models. Granulomas contain aggregations of immune cells, initially macrophages, and are formed by chronic mycobacterial stimulation (**Figure 1**). They range in size from a few mm to 20 mm (16, 17) and are round, ellipsoid, elongated or even branched (18, 19). Histopathology, immunohistochemistry and ultrastructural microscopy revealed the cellular composition of developing granulomas. As the disease progresses, monocyte-derived macrophages, dendritic cells and granulocytes, including neutrophils and eosinophils, are recruited proximally to the Mtb-infected macrophages. The cellular influx promotes granuloma biogenesis and macrophages within these structures rapidly progress toward epithelioid differentiation as shown in guinea pigs (14), mice (20), cattle (12), rabbits (15) and NHPs (13). These nascent granulomas increase in size and complexity. T- and B-cells surround the macrophage core of the mature TB granuloma. Non-immune cells including fibroblasts also contribute to granuloma architecture and maintenance (21, 22). The timing of immune cell afflux defines the architecture of the lung granuloma, including cellular neighborhoods and cell-cell communication. Lymphocytes enriched in the outer layer do not interact with the inner myeloid cell compartment due to spatial distance (23). This limits the mycobactericidal arsenal of the phagocytes (24, 25). Even within an individual, granulomas can have distinct immune signature (26, 27) and fates, ranging from sterilizing to caseating entities that facilitate Mtb dissemination (28, 29). Spatial mapping of individual immune cells revealed a superstructural histopathologic feature of the human TB granulomas (4) with distinct non-necrotizing cellular aggregates proximal to necrotizing granulomas. The fate of TB granulomas is determined by their cellular composition. An excess of neutrophils accompanies caseating granulomas and immunopathology (30, 31), whereas an early CD4 T-cell accumulation and local macrophage activation prevent intra-granulomatous necrosis (31).

Macrophages acquire distinct features that define the zonation of TB granulomas. Foamy and multinucleated giant cells (MNGCs) are distributed in different regions while epithelioid macrophages surround the core of Mtb-infected cells. Epithelization is driven by chronic mTORC1 stimulation (32) in combination with the IL-4/STAT6 signaling pathway which triggers E-cadherin expression on macrophages (33). In addition to the upregulation of genes regulating tight junctions and desmosomes, mainly E-cadherin seems critical for granuloma organization (34). This tight layer seals Mtb within the necrotic core and also limits drug penetration. Granuloma zonation has also been linked to macrophage polarization and to specific myeloid cell subpopulations. The M1 and M2 macrophage dichotomy fails to describe the continuum of macrophage activation, yet markers associated with these phenotypes occupy distinct granuloma niches. Anti-inflammatory (M2-like) macrophages enriched in arginase 1 (Arg1) versus inducible nitric oxide (iNOS) (35) are proximal to caseating foci within primate granulomas. Such patterns differ in other natural hosts. Porcine TB granulomas accumulate Arg1^+^ cells regardless of the lesion stage whereas iNOS-expressing M1-like cells populate bovine early-stage granulomas (36). Subsets of M2-like macrophages identified as CD16^+^CD163^+^MerTK^+^pSTAT3^+^ cells are associated with lesion severity in humans (37). iNOS/eNOS, Arg1 expression and additional factors, e.g. IL-10 signaling, drive regional macrophage polarization within TB granulomas. Subsets of neutrophils releasing neutrophil extracellular traps in a type I interferon (IFN-I)-dependent process are associated with central granuloma caseation (38). The early influx of IFN-I-producing plasmacytoid dendritic cells and interstitial macrophages may influence neutrophil function, and subsequent lesion zonation and outcome (39, 40). Fatty acid oxidation (FAO)-dependent neutrophils are recruited to granulomas (41) and cells with increased mitochondrial metabolism, similar to FAO-relying alveolar macrophages (42), support Mtb replication and induce immunopathology (43). Adaptive immunity in TB is mainly defined by B and T cells in the periphery of the TB granuloma. CD8+ T cells are essential in early control of TB in macaques (44). On the other hand, CD4+ T cells have a significant role in protection against reinfection (45). Granuloma-associated lymphoid tissue containing Mtb-specific B cells proximal to T follicular helper-like cells appear essential for disease control (46). Further diversity of CD3^+^ and CD8^+^ T-cell and B cell distribution within individual human TB granulomas was unveiled by quantitative multiplexed immunofluorescence which emphasizes presence of distinct immune environments (47).

High-resolution single cell analyses suggest further a zonation of granulomas into microenvironments and cellular ecosystems (**Figure 1**). Microenvironments with highly localized immunoregulatory programs, e.g. indoleamine 2,3-dioxygenase 1 (IDO1)- and PD-L1-expressing myeloid cells or proliferating regulatory T-cells, have been described (21). Variable multicellular ecosystems endow granulomas with Mtb restrictive functions (13). Bacilli persist in granulomas showing a type 2 immunity and wound healing programs. Mtb control requires niches enriched for type 1/type 17, stem-like, and cytotoxic T-cells. Spatial transcriptomics revealed regions containing SPP1^+^ macrophages distributed from the periphery to the granuloma center (48, 49). These engage with fibroblasts in bilateral cross-talks possibly contributing to lesion outcome. The spatial organization of TB granulomas follows pro- and anti-inflammatory mediators (50). Granuloma centers are rich in reactive oxygen species and pro-inflammatory eicosanoids whereas an anti-inflammatory signature surrounds the caseum. This physical segregation of the mediators may stem from various cellular niches. Thus, different cellular phenotypes and secretory products localize in specific areas, control bacilli growth and cell death events within TB granulomas.

### Zonation of gases, nutrients and metabolites

One of the key features of TB granulomas is a gradual decrease in oxygen levels towards the center (51, 52). Certain host species, including guinea pigs, rabbits, NHPs, and selected mouse strains, i.e. C3HeB/FeJ, exhibit hypoxic and well-defined granulomas (53, 54). Although organized granulomas were described in very-low dose (20) and ultra-low dose (55) infected C57BL/6 mice the oxygen abundances within remain unknown. Hypoxia promotes a non-replicating, persistent Mtb state that is also induced by nitric oxide (NO) and carbon monoxide (CO) gas gradients (56, 57). The hypoxic granuloma core is often necrotic, regardless of whether the infection is latent or active (58, 59). Disease exacerbation by necrosis is enhanced under hypoxia, leading to extracellular matrix destruction through the upregulation of collagenase and elastases (60).

Hypoxia-mediated inflammation is conserved across species (61). It triggers the stabilization of HIF-1α and the subsequent adaptation of immune cells to oxygen limitation. Depending on their spatial distribution, certain immune cell populations face oxygen deprivation within the TB granuloma. Hypoxia affects the architecture and dynamics of intracellular organelles relevant to defense against Mtb and these changes may be independent of HIF1-α stabilization (62–66). Nevertheless, HIF-1α stabilization reduces the influx of CD4^+^ and CD8^+^ T-cells, impairing Mtb control (67). Mtb produces d-serine under hypoxia, which hampers CD8-mediated IFN-γ secretion via dysregulated mTORC1 functions (68). Hypoxia related immune regulation and spatial reorganization within granulomas have also been demonstrated for innate immune cells including macrophages (69, 70), and neutrophils (71, 72). Abundances of macrophage Arg1 and neutrophil proteases, such as cathepsin G, are altered under hypoxia. The macrophage-rich core splits into two metabolic zones: a hypoxic, high-burden zone and an IDO^high^ zone that limits lymphocyte infiltration, weakening immunity in the NHP model (73). Thus, both innate and adaptive immune cells are affected by hypoxia in TB. Certain pro-angiogenetic factors such as vascular endothelial growth factor (VEGF), which are regulated by hypoxia, modulate granuloma architecture. While VEGF is highly abundant in the necrotic core and in surrounding macrophages (74) of human and rabbit (75) granulomas, microvessels are mainly present in the granuloma periphery and exhibit spatial and morphological heterogeneity (75). Propensity of macrophages to produce VEGF upon Mtb infection was shown with human cells (76). Inhibition of VEGF results in resolution of hypoxia, reduced inflammation, survival and vascular normalization in rabbits and mice (74, 75).

Changes within TB granulomas are not limited to oxygen levels. Mtb stimulates the expression of heme oxygenase 1 (HO-1) in macrophages which is highly abundant in the lungs of Mtb-infected mice (77). HO-1 produces CO, which remains elusive in the context of TB granulomas. Another gas, hydrogen sulphide (H_2_S) recently gained attention in TB. H_2_S is produced by several host enzymes including cystathionine β-synthase (CBS). H_2_S is a vasodilator that can cause inflammation via NF-κB activation (78). It may also negatively regulate iNOS and NO production (79). Mice deficient in CBS or treated with enzyme inhibitors survive longer with reduced bacterial load and lung inflammation (80). Despite these recent findings, there is still a knowledge gap about roles of these gases in pathophysiology of the TB granuloma.

Hypoxia is linked to glycolysis in the TB granuloma. Increased glycolysis triggers the formation of acidic by-products such as lactate which acidifies the lesion microenvironment in guinea pigs and mice (81–83). Lung tissue from human TB patients may reach acidic (pH ≤5.5) pH (84). The pH in rabbit caseum within granulomas increases from 6.4 to 7.4 upon lesion maturation (85), which is accompanied by changes in cellular composition. Thus, existence of zones with different pH within individual granulomas or within certain granuloma subtypes is plausible. On the other hand, *in situ* lesion pH in C3HeB/FeJ mice does not reach a pH below 7 (86).

Regarding the tricarboxylic acid cycle, the intermediate itaconate modulates host immunity in TB. Itaconate triggers anti-inflammatory responses, influences bacterial clearance, and affects tissue pathology in mouse models (87). Given the intracellular localization and limited cell permeability of itaconate, its potential influence on the microenvironment of TB granulomas requires further investigation.

Local hypoxia leads to a shift in the metabolic landscape beyond glycolysis. In humans, caseating granulomas are associated with increased lipid metabolism, especially cholesterol, cholesterol esters and triacylglycerols (88). Arginine and tryptophan are metabolized by the host via Arg1 or NOS, and IDO, respectively. Unavailability of Arg1 in the absence of iNOS leads to increased Mtb burden in hypoxic granulomatous structures (70). Epithelioid macrophages express higher levels of eNOS and iNOS than Arg1 with immunosuppressive macrophage phenotypes proximal to the lymphocyte cuff (35). Thus, the spatial availability of arginine may be critical for the pathophysiology of TB granulomas. The enzymes IDO1 and IDO2 perform the first-rate limiting step of L-tryptophan conversion to L-kynurenine and are highly expressed in macrophages within the granuloma. IDO^high^ macrophages are present in caseating granulomas in the NHP model (89). They may also interact proximally with T-cells and thus may be involved in the modulation of T-cell functions (90). In guinea pig granulomas, upregulation of many metabolites including amino acids such as alanine, glutamate and aspartate mimics metabolic changes in solid tumors (83). L-alanine promotes NF-κB activation and antimicrobial peptide production. Mtb expresses the alanine dehydrogenase Rv2780 which hydrolyzes the intracellular L-alanine pool and thereby suppresses the NF-κB-mediated expression of defensins in the murine TB model (91), but the relevance of this process to granuloma biology requires further investigation.

Metabolites of the arachidonic acid (AA) pathway are also critical for the pathophysiology of TB granulomas. These (e.g. products of ALOX5, ALOX5AP and LTA4H) are distributed throughout the granuloma with elevated levels in cavitary and caseous human granulomas (50). Within solid lesions, LTA4H is mainly distributed among MNGCs. In caseous and cavitary granulomas as well as alveolar inflammatory infiltrates, it is mainly distributed in macrophages/DCs and MNGCs (50). Caseous and cavitary granulomas show low levels of cyclooxygenases (COX1 and 2), which are responsible for the synthesis of prostaglandins (PGH2 and PGE2 or PGD2) (92). Since Mtb inhibits the eicosanoid pathway and negatively affects innate and adaptive immunity as well as cellular necrosis (93, 94), zonation of these metabolites affects granuloma stability.

In summary, the TB granuloma is a highly dynamic entity shaped by metabolic shifts, cell interactions, and oxygen deprivation. Metabolic adaptations further shape the immune landscape of granulomas, adding another layer of complexity (**Figure 1**). Because timing and location control the composition of the cellular microenvironment and Mtb permissiveness, a thorough understanding of the spatial immunobiology of the TB granuloma is essential for advancing the understanding of TB.

## Opportunities for therapies targeting TB granuloma biology

Standard TB chemotherapy consists of a combination of antibiotics over several months. Compliance is often poor due to the long duration of treatment and drug side effects (95), necessitating additional treatment options. Moreover, TB granulomas ‘wall off’ mycobacteria and also antibiotics. For example, *in situ* analysis of fluoroquinolones showed a variable distribution in immune cells within granuloma zones (96). Therefore, novel antibiotics should take granuloma biology into account, as lesion penetration is essential for good clinical outcomes. We focus on adjunctive HDTs in the context of pulmonary TB granulomas.

As diverse as granulomas in TB patients are, so are the requirements for HDTs (97). In the context of granuloma biology, several adjunctive HDTs are attractive (**Figure 1**). Type 4 phosphodiesterase inhibitors (PDE) such as CC-11050 exert their anti-inflammatory effects by limiting cAMP degradation. Increased cAMP levels lead to suppression of pro-inflammatory cytokines, prevention of inflammatory cell proliferation and slowing down of fibrotic processes (98). The downregulation of TNF-α networks by CC-11050 treatment is particularly relevant in TB (99, 100), given the role of this cytokine in the early formation and maintenance of granulomas (101, 102). Combined with antibiotics PDE inhibitors reduced the bacillary load in the lungs of rabbits and mice (99, 100) and improved TB outcomes in patients. CC-11050 completed a phase 2a clinical trial with increased lung recovery and a good preliminary safety profile (NCT02968927) (103). Moreover, CC-11050 and CC-3052 (another PDE inhibitor) modulated the wound healing networks in a rabbit model of TB (99, 104). Factors involved in this process were matrix metalloproteases (MMPs). MMPs include collagenases (e.g. MMP1 and MMP13), gelatinases (e.g. MMP2 and MMP9), stromelysins (e.g. MMP3), elastases (e.g. MMP12), and membrane-anchored proteases (e.g. MMP14) (105). They are involved in connective tissue homeostasis and fibrotic processes associated with TB granulomas. Excessive expression of MMPs in TB leads to lung tissue damage and cavitation. The use of an anti-MMP9 antibody as an adjunct to TB drugs reduced TB relapse rates (106). Hydroxamate-based compounds have a broad-spectrum activity against MMPs by mimicking collagen. Marimastat belongs to this class of drug and inhibited early granuloma formation besides reducing Mtb growth in a lung tissue model (107). Marimasat and Batimastat (a compound in the same class) in combination with anti-TB drugs reduced vascular leakage, stabilized blood vessels and improved drug penetration by targeting MMP2 and 9 (108). Doxycycline, an FDA-approved antibiotic and systemic MMP inhibitor (109) is being evaluated for off-target use in pulmonary (NCT02774993, NCT06477185) and central nervous system TB (NCT06446245). Besides bactericidal and sustained immunomodulatory effects via downregulation of IFN I and II, doxycycline reduces cavitary volume in the lungs of TB patients (109).

Granuloma formation is closely related to angiogenesis (27, 110). Normalization of Mtb-induced angiogenesis results in reduced bacillary growth and dissemination (110). Administration of losartan, an AT1 receptor antagonist, and bevacizumab, a human anti-VEGF monoclonal antibody, showed regularized blood vessels and extracellular matrix in granuloma microenvironment. These agents reduced Mtb burden in a rabbit model of TB alone and in combination with TB drugs (111). Delivery of an anti-VEGF antibody also resulted in reduced hypoxic regions and increased small molecule delivery (75). As discussed above, host enzymes associated with macrophage polarization control Mtb replication and the inflammatory milieu within the TB granuloma. Targeting the AA pathway seems amenable for the development of HDTs (112). Targeting IDO as adjunct to chemotherapy also showed promising results in NHP models (113). HDTs could balance the immune response within the granuloma considering its various microenvironments. Current solutions are limited to specific molecules or pathways, and many regulators of granuloma zonation remain to be targeted.

## Outlook

Multi-omics approaches in spatial biology currently advance the study of diseased tissues at unprecedented resolution. However, there is a growing need for novel *ex vivo* models to complement animal models that develop human-like TB granulomas. We envision that integrative approaches incorporating patient biopsies, animal samples, and *ex vivo* models will mutually facilitate a detailed understanding of the spatiotemporal cues of the TB granuloma.

Opportunities to advance knowledge of TB granulomas are provided by new technologies, high performance computing and modeling. Granuloma models based on computational methods (114, 115) and 3D spheroids (116, 117) facilitate studies of cellular responses under specific conditions such as hypoxia. Spheroids mimic *in vivo* granulomas, including the dormant state of Mtb (118, 119). Further opportunities include the integration of novel features into 3D models and/or *in silico* simulations. New experimental data link macrophage ontogeny (120), phenotype (enzymes, cell surface markers e.g. CD38) (121) and immunometabolism (FAO or glycolysis) to Mtb killing propensity, but the spatial context is still lacking. Similarly, clarifying the non-redundant roles of CD4^+^ and CD8^+^ lymphocyte subsets (44, 45) or positioning of granuloma-associated lymphoid tissue (46) in the spatial context could help define correlates of protection. In this respect, precise localization and interactors of B cell clusters including plasma cells, as detected in NHPs (122), and zonation of Mtb-specific antibodies remain elusive. The TB granuloma has already been mathematically modeled (123). Refinements and AI implementation to integrate multi-omics could help predict granuloma trajectories. New *in vivo* tracers of inflammation (124) and AI-based imaging algorithms could predict responses to therapies and accelerate HDTs.

The challenges in determining granuloma heterogeneity lie in the diverse lifestyle of Mtb. In addition to its intracellular and extracellular localization, Mtb’s subcellular lifestyle adds a layer of complexity and modulates host responses. Visualization of the subcellular localization of Mtb within infected tissue using correlative light electron microscopy is feasible, but limited to a few specialized laboratories and precludes high-throughput scale (125). Drug penetrance is conditioned by subcellular niches (126), and thus uncovering microenvironments with distinct subcellular Mtb residency is relevant for TB chemotherapy. Mtb genetic diversity influences disease severity and lung pathology (127), and must be considered. Attempts to model granuloma responses to distinct Mtb lineages (128) encourage further studies aiming to elucidate the extent to which Mtb lineage alters zonation or cellular niches.

Singular HDT approaches are currently being tested, but future therapy of TB patients could be guided by clinical data and individualized. Complex diseases require personalized therapies, and perhaps TB endotypes (129) are based on granuloma variability. Embracing heterogeneity at the microenvironmental level paves the way for exciting discoveries about granuloma biology and TB therapy.
